# Supplementary material for: Host Cell Metabolism Contributes to Delayed-Death Kinetics of Apicoplast Inhibitors in Toxoplasma gondii
Source: Antimicrob Agents Chemother. 2019 Jan 29;63(2):e01646-18. doi: 10.1128/AAC.01646-18 (PMC6355570; doi:10.1128/AAC.01646-18)
Supplement: Supplemental file 2 [file df3fac710ea23106b9940116cb9012f0_AAC.01646-18-s0002.pdf]

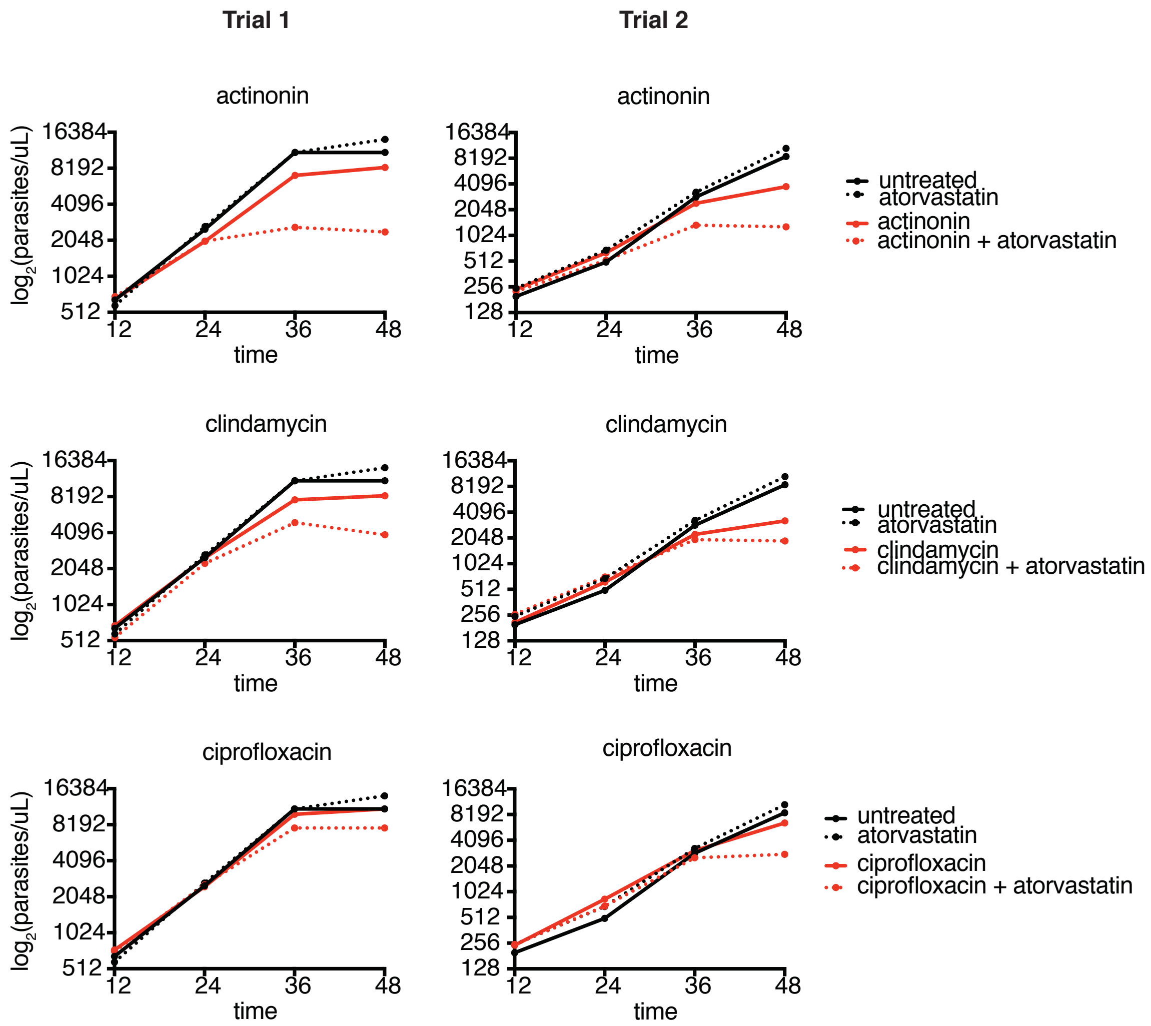

Figure S1: Raw growth curves demonstrate that co-treatment with atorvastatin and apicoplast inhibitors leads to earlier growth defects than treatment with either inhibitor alone. Parasite growth quantified by flow cytometry of *T. gondii* manually released from host cells at each time point after treatment with or without atorvastatin or apicoplast inhibitors. Each biological replicate is plotted separately with the respective controls from that experiment.

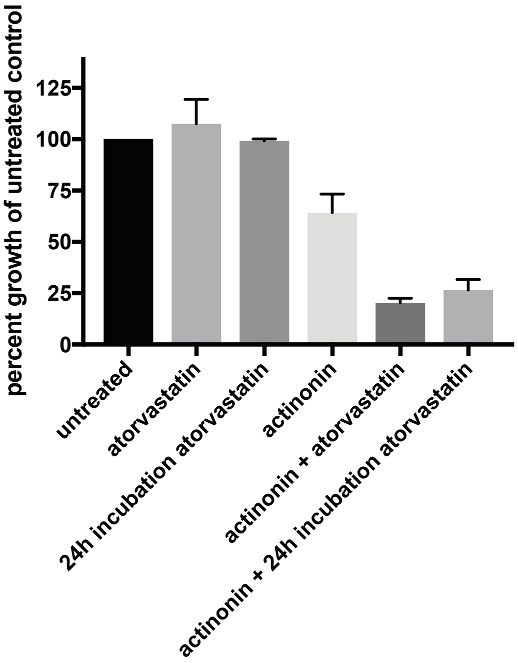

Figure S2: Pre-incubation of host cells with atorvastatin for 24 hours prior to infection does not exacerbate growth defect of cells co-treated with atorvastatin and apicoplast inhibitors. 1.5 million parasites were allowed to infect T25 flasks containing confluent human foreskin fibroblasts (HFFs) that were either untreated or pre-incubated with atorvastatin for 24 hours prior to infection. Infected cells were then incubated with either no inhibitor, atorvastatin only, actinonin only or actinonin + atorvastatin. Parasite growth at the end of a 48 hour lytic cycle was quantified by flow cytometry. Results are from 2 biological replicates (actinonin + 24h incubation atorvastatin) and ≥2 biological replicates (all other conditions). Error bars represent the standard error of the mean (SEM).
